# Understanding Racial and Rural Disparities in the Relationship between Social Isolation and Social Technology Use

**DOI:** 10.1093/geroni/igab046.3351

**Published:** 2021-12-17

**Authors:** Kaileigh Byrne, Reza Ghaiumy Anaraky, Hannah Barfield, Summerlin Nickel

**Affiliations:** Clemson University, Clemson, South Carolina, United States

## Abstract

Social isolation is characterized by lack of social contacts and high degrees of loneliness. Feelings of loneliness and social isolation are linked to declines in cognitive functioning and increased risk of dementia. Previous research suggests that loneliness is more prevalent among Black and rural older adults compared to White and urban-dwelling older adults. Given these disparities, it is important to identify methods that reduce social isolation and loneliness among this population. Social technology, such as Facebook and Skype, is one possible way to connect with others. This study uses the Health and Retirement Study (HRS) dataset to examine racial and rural disparities in the relationship between social technology use and social isolation, loneliness, and social support among individuals age 50 and older. The overarching hypotheses are that (1) rural-dwelling older adults and older Blacks will report less social technology use compared to urban-dwelling and older White adults, and (2) there will be a negative relationship between loneliness and social technology use, and (3) a positive relationship between perceived positive social support and social technology use. Racial or rural disparities in these latter potential relationships are exploratory. Multiple linear regression analysis will be performed to assess these relationships. Preliminary correlational results indicate that, consistent with prior work, greater use of social technology was associated with higher social support (N=6,029; r=.29, p<.001). However, contrary to our hypothesis, greater self-reported loneliness was associated with greater social technology (r=.09, p<.001). Examination of potential racial and rural disparities in these relationships are currently underway.

